# Enhancing Endosomal Escape for Intracellular Delivery of Macromolecular Biologic Therapeutics

**DOI:** 10.1038/srep32301

**Published:** 2016-09-08

**Authors:** Peter Lönn, Apollo D. Kacsinta, Xian-Shu Cui, Alexander S. Hamil, Manuel Kaulich, Khirud Gogoi, Steven F. Dowdy

**Affiliations:** 1 Dept of Cellular and Molecular Medicine, UCSD School of Medicine, La Jolla, CA, 92093 USA

## Abstract

Bioactive macromolecular peptides and oligonucleotides have significant therapeutic potential. However, due to their size, they have no ability to enter the cytoplasm of cells. Peptide/Protein transduction domains (PTDs), also called cell-penetrating peptides (CPPs), can promote uptake of macromolecules via endocytosis. However, overcoming the rate-limiting step of endosomal escape into the cytoplasm remains a major challenge. Hydrophobic amino acid R groups are known to play a vital role in viral escape from endosomes. Here we utilize a real-time, quantitative live cell split-GFP fluorescence complementation phenotypic assay to systematically analyze and optimize a series of synthetic endosomal escape domains (EEDs). By conjugating EEDs to a TAT-PTD/CPP spilt-GFP peptide complementation assay, we were able to quantitatively measure endosomal escape into the cytoplasm of live cells via restoration of GFP fluorescence by intracellular molecular complementation. We found that EEDs containing two aromatic indole rings or one indole ring and two aromatic phenyl groups at a fixed distance of six polyethylene glycol (PEG) units from the TAT-PTD-cargo significantly enhanced cytoplasmic delivery in the absence of cytotoxicity. EEDs address the critical rate-limiting step of endosomal escape in delivery of macromolecular biologic peptide, protein and siRNA therapeutics into cells.

Bioactive macromolecules, including peptides, proteins and siRNAs, possess many desirable therapeutic features that provide unique opportunities to design precision medicine therapeutics to treat human disease. However, due to their size (>1,000 Da), macromolecules have no bioavailability to cross the cell membrane and enter cells, and therefore, require the use of an efficient delivery agent to access their site of action in the cytoplasm or nucleus^1^,^2^. The serendipitous identification of a basic cationic delivery peptide from the HIV TAT protein in the late 1980’s^3^,^4^, called a peptide/protein transduction domain (PTD)^5^ or cell penetrating peptide (CPP)^6^, paved the way to delivery of macromolecular therapeutics. The TAT PTD has now been widely used to transport a variety of macromolecules into a plethora of cell types, as well as pre-clinical models of disease and multiple clinical trials, including a statistically significant phase-II and an ongoing phase-III trial^1^,^7^. Thus, the TAT PTD has enabled the development of entirely new classes of intracellular molecular treatments^1^,^2^,^8^,^9^.

TAT PTD and related PTDs/CPPs deliver macromolecules into cells by endocytosis^1^,^2^,^8^,^9^,^10^,^11^. In 2004, we designed a rigorous live cell phenotypic transduction assay to further delineate the endosomal pathway(s) used by PTD/CPPs^11^. Using a cell based phenotypic assay based on TAT-Cre delivery and recombination of genomic DNA to induce GFP expression^11^, we showed that the TAT PTD mediates cellular delivery by performing two critical processes: 1) TAT PTD actively stimulates it’s own uptake by induction of macropinocytosis, a specialized form of endocytosis, and 2) TAT PTD undergoes endosomal escape. Importantly, endosomal escape is still hands-down the rate-limiting step for delivery of macromolecules into the cytoplasm^11^,^12^,^13^,^14^. While the TAT-Cre cellular uptake assay as well as other phenotypic transduction methods have greatly aided with dissecting the cellular transduction mechanism, there has been a lack of a suitable live cell quantitative phenotypic transduction assay with a low to zero false-positive rate to effectively dissect escape into the cytoplasm. Development of such assays would help address important questions regarding quantification of uptake, routes and dynamics of internalization, as well as how to improve the design of next-generation delivery vehicles and endosomal escape domains for macromolecular biologic therapeutics.

Recently several groups^15^,^16^ have used bi-molecular split-GFP fluorescence auto- complementation^17^,^18^,^19^ assays to detect delivery of various cargo into the cytoplasm of live cells.

GFP is composed of 11 β-strands that form a barrel structure allowing for peptidyl backbone cyclization and formation of a fluorescent chromophore^20^. Cabantous *et al.* showed that removal of the 16 residue β-strand #11 (GFPβ11) (AA #215–230; RDHMVLHEYVNAAGIT; 1,826 Da) from an optimized superfolder GFP molecule results in a large non-fluorescent GFP fragment (GFPβ1-10) (residues 1–214)^17^,^18^,^19^. However, co-incubation of the large, non-fluorescent GFPβ1-10 fragment with the GFPβ11 peptide *in trans* efficiently reconstitutes the GFP fluorescent chromophore bond (backbone peptidyl cyclization) and restores GFP fluorescence.

We reasoned that the live cell spilt-GFP complementation phenotypic transduction assay offers several important advantages for monitoring PTD/CPP transduction of macromolecular cargo into cells: 1) the GFPβ11 peptide is too large to enter cells alone and requires a delivery domain and endosomal escape agent to enter the cytoplasm, resulting in a zero false-positive rate from peptides stuck on the cell surface or trapped in endosomes, 2) the transduction process and escape into the cytoplasm can be quantitatively monitored in real-time by flow cytometry (FACS) for GFP complementation fluorescence, and lastly 3) unlike signal amplifying indirect measuring assays, such as the TAT-Cre recombinase^11^ or splice correction assays^21^ that do not directly correlate with the number of macromolecules delivered inside of cells, transduction of GFPβ11 by PTDs/CPPs will induce GFP fluorescence at a 1:1 ratio that allows for a direct quantitative measurement of GFPβ11 peptides that have escaped the endosomes and are present in the cytoplasm. Moreover, the assay is technically simple and only requires synthesis of the GFPβ11-TAT peptide, thereby aiding with ease of use

Using the split-GFP complementation assay with TAT-PTD/CPP,, we investigated an array of endosomal escape domains (EEDs) with various hydrophobic features (Fig. 1a). Hydrophobic residues have previously been described to play important roles in endosomal escape of viruses and are also known to modulate PTD/CPP uptake^11^,^22^,^23^,^24^,^25^. Furthermore, we also investigated the effect of the position of EEDs in relation to the TAT PTD/CPP. We found that the addition of EEDs with specific hydrophobic patches, containing either two aromatic indole rings or one indole ring and two aromatic phenyl groups, at a fixed distance of six polyethylene glycol (PEG) units from the PTD-cargo significantly enhanced cytoplasmic delivery in the absence of cytotoxicity. EEDs are an exciting addition that opens up new potential for intracellular delivery of new macromolecular biologic therapeutics.

## Results

### Design of real-time, live cell quantitative split-GFP fluorescence transduction assay

First we synthesize a GFPβ11-TAT peptide and investigated if it would complement with GFPβ1-10. *In vitro* mixing of the synthetic GFPβ11-TAT peptide with purified GFPβ1-10 protein fragment at 37 °C resulted in a steady time-dependent increase in GFP fluorescence that started to plateau at 1 h and reached maximal GFP fluorescence by 2–4 h (Fig. S1).

Next, we administered GFPβ11-TAT to cells stably expressing the GFPβ1-10 fragment to examine if we can quantitatively determine the cytoplasmic uptake by PTDs/CPPs in live cells via restoration of GFP fluorescence. We generated stable cell clones constitutively expressing the non-fluorescent large GFPβ1-10 fragment, including H1299 non-small cell lung carcinoma, HaCaT immortalized keratinocytes, and MDA-MB-231 and MCF7 breast carcinomas. Treatment of GFPβ1-10 expressing human H1299 cells with increasing concentration of GFPβ11-TAT peptide (0–60 μM) resulted in a robust intracellular GFP fluorescence complementation signal (Fig. 2a,b; Fig. S2a–d). The near linear dose-dependent increase in fluorescence suggested an absence of a critical threshold concentration for intracellular uptake and endosomal escape. Interestingly, FACS histogram analysis revealed that most, if not all, individual cells displayed GFP fluorescence complementation and hence, were transduced (Fig. 2b). In contrast, addition of control GFPβ11 peptide plus TAT peptide *in trans* (non-conjugated) failed to induce GFP fluorescence above background (Fig. 2a,b). Likewise, treatment of parental control H1299 cells (no GFPβ1-10 fragment) with GFPβ11-TAT peptide failed to increase fluorescence above background (Fig. S2d). Furthermore, GFPβ11-TAT peptide treated cells did not display any cytotoxicity or morphological changes (Fig. 2c,d). Similar dose-dependent results were obtained after addition of GFPβ11-TAT to three additional GFPβ1-10 expressing cell types, MCF7, MDA-MB-231 and HaCaT cells (Fig. S2d–i). Consistent with an actin-dependent macropinocytotic endocytosis uptake mechanism^11^,^12^, RNAi knockdown of Rac-1 or treatment with the macropinocytosis inhibitor, EIPA, resulted in reduced GFP fluorescence to near background levels (Fig. S3).

We next examined the kinetics of delivery. H1299 GFPβ1-10 expressing cells were treated with GFPβ11-TAT peptide for various amounts of time. We observed GFP fluorescence as early as 20 min with a steady increase of signal that plateaued after 2 hr (Fig. 2e,f). Fluorescent video microscopy of GFPβ11-TAT peptide treated cells confirmed the time-dependent increase in GFP fluorescence throughout the entire cell population (Fig. S4). Thus, taking GFP chromophore maturation kinetics into account (Fig. S1), these experiments showed that TAT-PTD-mediated uptake is occurring rapidly after addition to cells and that the majority of uptake and escape into the cytoplasm is likely complete within the first hour. Collectively, these observations validated the utility of bimolecular split-GFP complementation as a live cell quantitative phenotypic delivery assay for measuring GFPβ11-TAT peptide delivery and endosomal escape into the cytoplasm with a zero false-positive rate.

### Hydrophobic endosomal escape domains

Successful delivery of macromolecules into the cytoplasm of cells requires three critical steps: 1) cell association, 2) stimulation of endocytosis, and 3) facilitation of endosomal escape. Of these, it is well appreciated that the critical bottleneck is escape of macromolecules from endosomes into the cytoplasm in a non-cytotoxic fashion^1^,^2^,^10^,^11^,^12^,^13^,^14^. Indeed, treatment of GFPβ1-10 expressing H1299 cells with a low concentration of a disulfide conjugated GFPβ11-(S-S)-TAT peptide plus Chloroquine, an endosomal disruption agent, resulted in a ~4-fold increase in GFP fluorescence (Fig. 3a). This observation confirmed that the bulk of GFPβ11-(S-S)-TAT peptide remained trapped in endosomes and also demonstrated the ability of the split-GFP assay to detect additional endosomal escape. Unfortunately, Chloroquine, as well as other endosomal escape or endolytic agents, are often too toxic for use in preclinical models or eventual clinical trials of macromolecular therapeutics. Alternatively, viruses have evolutionarily addressed the endosomal escape problem by destabilizing the endosomal lipid bilayer membrane by insertion of motifs containing hydrophobic amino acid R groups^22^. Previously, we had used the hemagglutinin (HA2) endosomal escape domain from influenza virus to enhance TAT-Cre protein delivery into cells^11^. Likewise, two groups, Dr. Futaki’s in Japan and Dr. Norden’s in Sweden, have previously shown that addition of hydrophobic aromatic ring containing amino acids, Phe (F) or Trp (W), can enhance PTD/CPP delivery peptides^23^,^24^,^25^.

Using the spilt-GFP assay, we systematically investigated how to enhance endosomal escape by covalent attachment of hydrophobic Endosomal Escape Domains (EEDs). To avoid any potential steric or hydrophobic interference between different EEDs and GFPβ11 complementation with cytoplasmic GFPβ1-10, we conjugated all GFPβ11 peptides to TAT-EED peptides via a disulfide linker that allows for intracellular reductive separation of the GFPβ11 cargo from the EED and TAT delivery domain. All disulfide conjugated peptides were purified by HPLC and quality controlled by mass-spectrometry. Both GFPβ11-TAT and GFPβ11-(S-S)-TAT peptides induced GFP fluorescence in a similar dose-dependent manner (Fig. S5). We first synthesized an EED containing two Tryptophan residues flanked by Glycine moieties for free bond rotation -GWWG on the C-terminus of GFPβ11-(S-S)-TAT (Fig. 3b,c). Treatment of GFPβ1-10 expressing H1299 cells with GFPβ11-(S-S)-TAT-GWWG resulted in a strong enhanced endosomal escape and GFP fluorescence; however, it simultaneously strongly increased cytotoxicity (Fig. 3c–e). In line with this, prior research on PTD/CPP transduction also showed that inclusion of fluorescent dyes (which are hydrophobic) or other hydrophobic residues may result in altered PTD peptide uptake and increased cytotoxicity compared to PTDs/CPPs alone^11^,^23^,^24^,^25^,^26^. Consequently, while aromatic residues enhanced endosomal escape, they did so at the expense of significantly increased toxicity, thereby limiting their use.

We reasoned that part of the basis of the cytotoxicity arose due to the close proximity of the hydrophobic EED to the charged TAT delivery domain. Therefore, we increased the distance between the EED and delivery domain by inclusion of a polyethylene glycol (PEG) molecular spacer. PEG is a hydrophilic, non-ionic, biologically inert polymer that is commonly used to improve the formulation and deliverability of various drugs^27^. We generated GFPβ11-(S-S)-TAT-PEG(n)-GWWG delivery domains with an increasing number of PEG units between TAT and the hydrophobic EED motif (Fig. 3b). Surprisingly, inclusion of a six PEG unit (P6) spacer in GFPβ11-(S-S)-TAT-PEG6-GWWG retained the enhanced cytoplasmic delivery, but significantly reduced the cellular toxicity, even at the highest concentration tested (60 μM) (Fig. 3c–e). However, increasing the spacer distance to 12 or 18 PEG units substantially lowered uptake or escape efficiency. Based on these results, we performed all subsequent experiments using the six PEG unit (P6) spacer.

### Screening hydrophobic endosomal escape domains

Both Trp (W) and Phe (F) residues are both known to destabilize cellular membranes by burying their hydrophobic R groups into the lipid bilayer^27^,^28^. To optimize the EED, we systematically synthesized C-terminal hydrophobic EEDs with various combinations of Trp and Phe residues that included the optimal six PEG unit (P6) spacer. After initial analyses, we focused our efforts on seven different hydrophobic EED motifs: -GFFG, -GWG, -GFWG, -GFWFG, -GWWG, -GWGGWG, and -GWWWG and a control –GG motif (Fig. 4a). Addition of aromatic rings from either two Phe residues, GFPβ11-(S-S)-TAT-P6-GFFG, or one Trp residue, GFPβ11-(S- S)-TAT-P6-GWG, to the C-terminus, had no net effect on delivery compared to the parental GFPβ11-(S-S)-TAT peptide (Fig. 4b–e). However, addition of aromatic rings from both a Phe and Trp, GFPβ11-(S-S)-TAT-P6-GFWG, showed a two-fold increase in GFP fluorescence compared to the parental GFPβ11-(S-S)-TAT peptide with no signs of cytotoxicity. Moreover, increasing hydrophobicity by inclusion of either Phe-Trp-Phe residues, GFPβ11-(S-S)-TAT-P6-GFWFG, or two Trp residues, GFPβ11-(S-S)-TAT-P6-GWWG, to the C-terminus, resulted in a five-fold increase in GFP fluorescence in the absence of cytotoxicity (Fig. 4b–e). However, addition of six aromatic rings by inclusion of three Trp residues, GFPβ11-(S-S)-TAT-P6-GWWWG, resulted in a dramatic increase in cytotoxicity that hampered uptake (Fig. 4b–e).

Increasing the spacing between the two Trp residues by insertion of two Gly residues, -GWGGWG in GFPβ11-(S-S)-TAT-P6-GWGGWG, decreased the enhancement significantly compared to the -GWWG motif, suggesting that a concentrated hydrophobic patch is required for the enhanced endosomal escape. We also noted that addition of a control C-terminal PEG6-GG tail, GFPβ11-(S-S)-TAT-P6-GG, resulted in a lower uptake compared to parental GFPβ11-(S-S)-TAT peptide, suggesting that a free PEG polymer tail alone reduced uptake. Surprisingly, while both the -GWWG and -GFWFG domains enhanced cytoplasmic escape compared to parental GFPβ11-(S-S)-TAT peptide, inclusion of four consecutive aromatic ring Phe residues, -GFFFFG, resulted in adverse cytotoxic effects on cells, causing gross morphological changes and cell death (Fig. S6), suggesting that too long of a hydrophobic patch results in cell membrane destabilization leading to cytotoxicity. TAT peptides are taken up into cells by stimulating macropinocytosis^11^,^12^. Using 70 kDa neutral dextran-Texas Red as a marker of macropinocytosis^11^, we determined that the control TAT, TAT-P6-GFWFG and TAT-P6-GWWG peptides all stimulate macropinocytosis to a similar extent (Fig. S7), arguing that the increased GFP fluorescence by GFPβ11-(S-S)-TAT-P6-GFWFG and GFPβ11-(S-S)-TAT-P6-GWWG peptides is indeed due to enhanced endosomal escape and not merely an increased stimulation of macropinocytosis. Lastly, we confirmed that the optimized GFPβ11-(S-S)-TAT-P6-GFWFG peptide significantly enhanced endosomal escape in three additional human cell lines in a non-cytotoxic fashion compared to the parental GFPβ11-(S-S)-TAT peptide (Fig. 5), suggesting that EEDs universally enhance endosomal escape.

## Discussion

Delivery of macromolecular cargo, including peptides, proteins and siRNAs, into cells and tissues has great potential as truly precision medicine therapeutics to treat human disease. To improve uptake, we and others have previously incorporated various hydrophobic domains to modulate function and uptake of PTDs/CPPs^11^,^23^,^24^,^25^,^26^; however, associated cytotoxicity has remained a significant problem. In this study, we employed a simple real-time, quantitative live cell phenotypic PTD/CPP transduction assay using a split GFP peptide cargo complementation approach to measure transduction of cargo in the cytoplasm. We used this assay to 1) examine if increasing the distance between the PTD/CPP and a hydrophobic EED could decrease the cytotoxicity but maintain cytoplasmic delivery, and 2) screen a selection of EEDs with increasing hydrophobicity. We found that a six PEG unit spacer (18 carbon bond lengths) resulted in an optimal separation distance between the PTD/CPP and the hydrophobic patch. This spacer helped maintain enhanced cytoplasmic delivery, but minimized cytotoxicity. Using a systematic approach, we then narrowed down the optimal EED composition to containing two indole rings or one indole ring and two phenyl groups in either a FWF or WW composition. Interestingly, Li *et al.* identified a highly charged Aurein 1.2 peptide that enhances endosomal escape 5-fold^29^.

**Figure 1:**
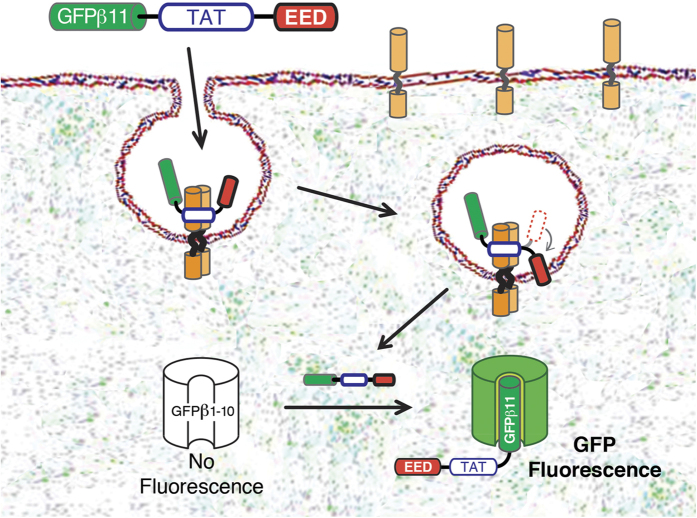
The study concept. PTD/CPP binds to negatively charged molecules on the cell surface and stimulates macropinocytotic uptake and endosomal escape of GFPβ11-PTD/CPP peptide into the cytoplasm. When concentrated with the PTD/CPP in the endosomes, the hydrophobic EED motif buries itself into the lipid bilayer membrane which leads to a localized membrane destabilization that enhances endosomal escape into the cytoplasm. Binding of GFPβ11 peptide to non-fluorescent GFPβ1-10 protein fragment in the cytoplasm induces chemical formation of the GFP fluorescent chromophore.

**Figure 2:**
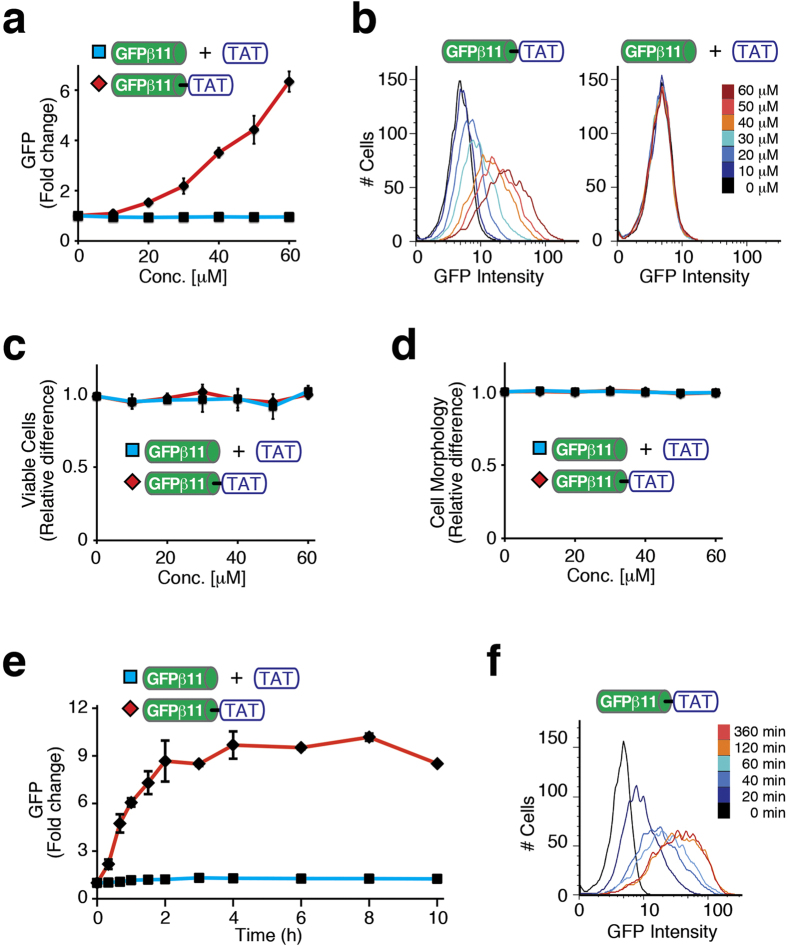
Transduction of GFPβ11-TAT induces fluorescence complementation of intracellularly expressed GFPβ1-10 protein fragment. (**a**) Dose-dependent comparison of GFPβ1-10 expressing H1299 human lung adenocarcinoma cells treated with GFPβ11-TAT peptide, or control GFPβ11 peptide plus TAT peptide (*in trans*) analyzed by FACS. The graph shows mean values of triplicate samples with S.D. (**b**) Histograms of GFPβ1-10 expressing H1299 cells treated with increasing doses of GFPβ11-TAT peptide, or control GFPβ11 peptide plus TAT peptide (*in trans*) analyzed by FACS. (**c,d**) Cell viability and morphology (FSC/SSC) of GFPβ1-10 H1299 cells treated with increasing doses of GFPβ11-TAT peptide or control GFPβ11 peptide plus TAT peptide. The graphs show mean values of triplicate samples with S.D. (**e**) Kinetic analysis of GFPβ1-10 H1299 cells treated with 40 μM GFPβ11-TAT peptide and control GFPβ11 peptide plus TAT peptide over time. The graph shows mean values of triplicate samples with S.D. (**f**) Histogram of GFPβ1-10 H1299 cells treated with GFPβ11-TAT peptide and control untreated and measured by FACS over time.

**Figure 3:**
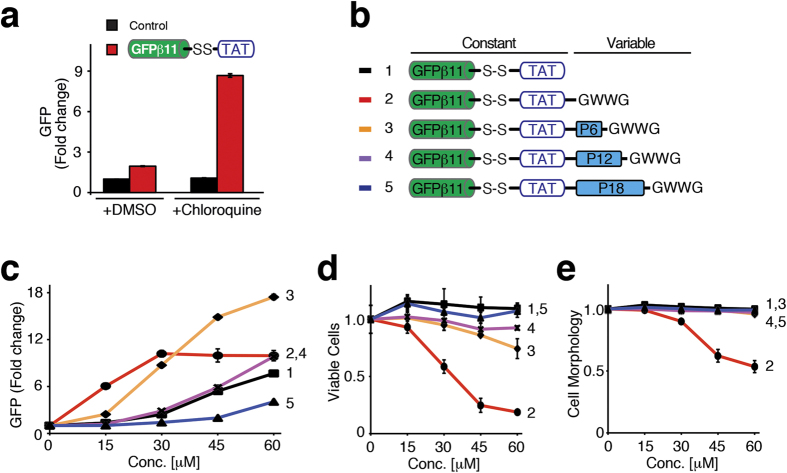
Optimizing endosomal escape by introducing PEG-spacers between PTD/CPP delivery domain and a hydrophobic patch. (**a**) GFPβ1-10 H1299 cells treated with GFPβ11-TAT peptide or untreated control were also treated with plus 100 μM Chloroquine, an endosomal disruption agent, and assayed for GFP fluorescence by FACS. (**b–e**) Dose-dependent comparison of GFPβ1-10 expressing H1299 cells treated with GFPβ11-(S-S)-TAT-PEG(n)-GWWG (**b**) peptides containing varying length (n) of PEG spacer (P) analyzed for GFP fluorescence (**c**), cellular morphology (**d**), and number of viable cells (**e**) by FACS. The graphs show mean values of triplicate sample analysis with S.D.

**Figure 4:**
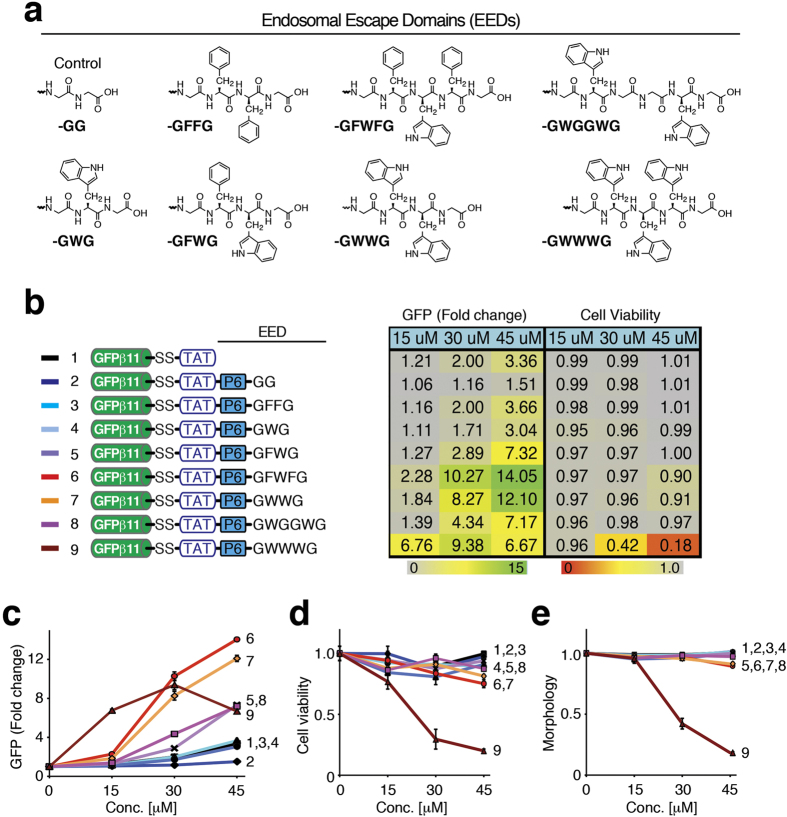
Optimizing design of endosomal escape domain (EED). (**a**) Structures of EEDs. (**b–e**) Dose-dependent comparison of GFPβ1-10 H1299-c#G3 cells treated with GFPβ11-(S-S)-TAT-(X) peptides containing a PEG6-spaced aromatic ring hydrophobic endosomal escape domain (EED), as indicated, to parental GFPβ11-(S-S)-TAT peptide and control GFPβ11-(S-S)-TAT-PEG6-GG peptide analyzed by FACS for GFP fluorescence (**b**,**c**), cell viability (**d**), and cellular morphology (**e**). The table (**b**) displays mean values from triplicate samples and the graphs (**c–e**) show the same mean values with S.D.

**Figure 5:**
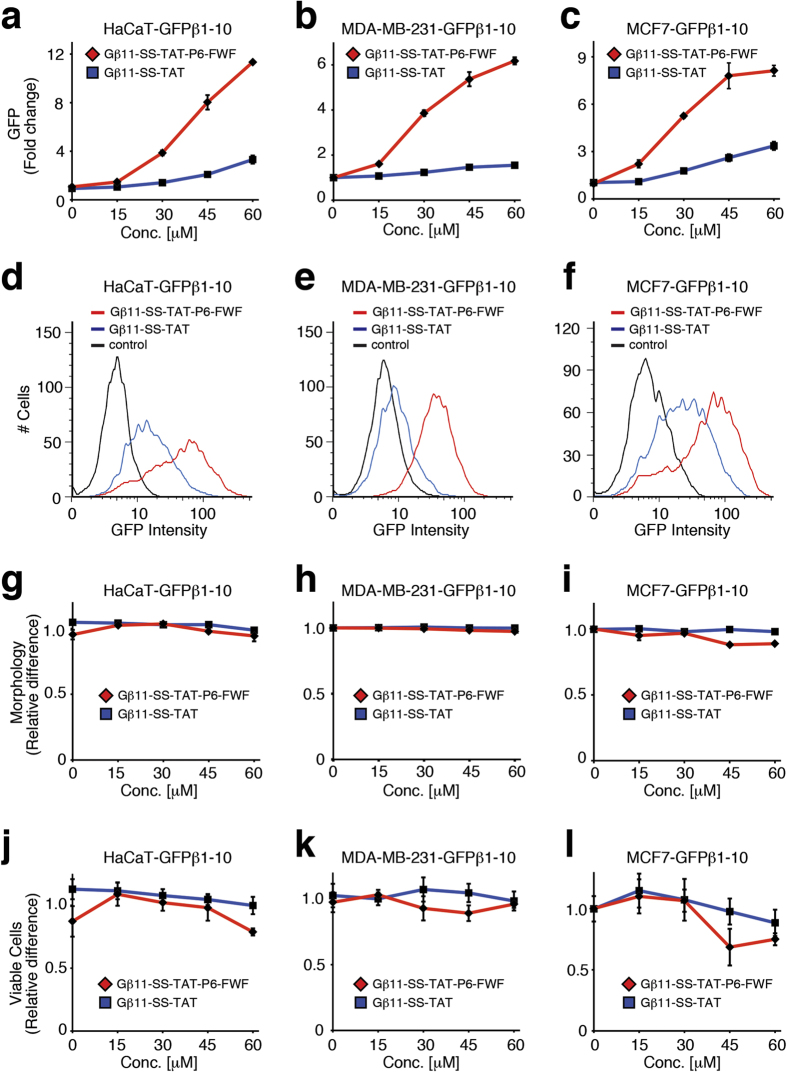
Evaluation of GFPβ11-(S-S)-TAT-PEG6-GFWFG peptide in multiple cell types. Dose-dependent analysis of GFPβ1-10 expressing HaCaT keratinocytes, MDA-MB-231 and MCF7 breast carcinoma cells treated with GFPβ11-(S-S)-TAT-PEG6-GFWFG peptide and parental GFPβ11-(S-S)-TAT peptide by FACS for GFP complementation fluorescence (**a–f**), cellular morphology (**g–i**) and cell viability (**j–l**), respectively. Graphs display mean values of triplicate samples with S.D.

We speculate that the improved PTD/CPP-EED domains developed here enhance cellular macromolecular delivery by insertion of the hydrophobic patch into the lipid bilayer at a critical distance (18 bonds) from the delivery domain. Thus, when concentrated in endosomes, the PTD/CPP-EED results in a strong localized membrane destabilization, leading to enhanced escape into the cytoplasm (Fig. 1a). However, further delineating of the exact mechanism(s) of action will require extensive biophysical studies. In conclusion, the TAT-EEDs described here show a significantly improved uptake profile compared to TAT alone and have potential to address the critical rate-limiting endosomal escape step in intracellular delivery of macromolecular biologic peptide, protein and siRNA therapeutics and to shape next-generation endosomal escape domains.

## Methods

### Plasmids, antibodies, siRNAs and other reagents

Mammalian optimized pCMV-mGFPβ1-10 plasmid (22004005) was purchased from Sandia Biotech. EIPA, Chloroquine, and DMSO were from Sigma. Anti-GFP (Invitrogen) and anti-α-Tubulin (Sigma) were used for immunoblotting. siRNA targeting human RAC1 (ID: s11711) and control siRNA (4611G) was bought from Ambion. Lipofectamine 2000 was purchased from Invitrogen.

### Cell culture, transfections and immunoblot analysis

H1299, MCF7, MDA-MB-231 and HaCat cells were maintained in DMEM supplemented with 10% FBS, 100 U/ml penicillin, and 100 U/ml streptomycin. H1299c#G3, H1299c#G4, MCF7c#G7, MDA-MB-231c#G3 and HaCaTc#G7 cells were generated by transfecting cells with pCMV-mGFPβ1-10 and subsequently grown under hygromycin selection. Hygromycin resistant cells were then treated with GFPβ11-TAT and individual, transiently fluorescent clonal cells were isolated by FACS sorting. Clones were expanded and tested for stable GFPβ1-10 expression. H1299c#G3, H1299c#G4, MCF7c#G7, MDA-MB-231c#G3 and HaCaTc#G7 cells were maintained in DMEM supplemented with 10% FBS, 100 U/ml penicillin, 100 U/ml streptomycin and 50 or 100 ug/ml hygromycin. Transient transfections of siRNA or plasmid DNA were performed using Lipofectamine 2000 according to manufacturer’s instructions. Immunblots were performed using 10% SDS-PAGE, semi-dry transfer (BioRad) and developed on ChemiDoc Imager (BioRad).

### Peptide synthesis

Fmoc solid phase peptide synthesis was performed using a Symphony Quartet peptide synthesizer (Ranin) and rink-amide MBHA resin as solid support. Protected amino acids and coupling reagents were purchased from Anaspec. Synthesized peptides were cleaved and deprotected using standard conditions (95% TFA with water and TIS) and subsequently precipitated using cold diethylether. Prep-scale RP-HPLC with an Agilent Prep C18 (30 × 250) mm column was used to for purification and peptide purity and size was confirmed by mass spectrometry using α-CHCA matrix (Voyager, Applied Biosystems DE-Pro MALDI-TOF). Peptides were then lyophilized and resuspended in pure water or in pure water with 5% glycerol and stored at −20 °C for short term or at −80 °C for long term.

### Disulfide conjugation

GFPβ11-Cysteine was combined with NPyS protected Cystein-PTD/CPP at 1:1.5 or 1.5:1 ratio. pH was adjusted to ~7.5 using PBS or Tris-HCl. Reactions were incubated 1 hr at RT before being purified using HPLC. Conjugation and purity of products was confirmed by mass spectrometry using α-CHCA matrix (Voyager, Applied Biosystems DE-Pro MALDI-TOF). Conjugated peptides were lyophilized and resuspended in water with 5% glycerol and stored at −20 °C for short term or at −80 °C for long term.

### *In vitro* complex formation of GFPβ11-TAT and GFPβ1-10

GFP-β11-TAT peptide was incubated with GFP-β1-10 protein for the indicated time-points in PBS on a black opaque 96-well plate at 37 °C. The plate was analyzed for GFP fluorescence using an IVIS Spectrum imager.

### Peptide transduction

All transduction experiments were performed in 48-well plates. An optimized protocol was established. First, 15,000 or 20,000 cells were plated in each well. Next day, the indicated peptides were pipetted into microcentrifuge tubes. Transduction buffer (60% OptiMEM and 40% PBS) was added to peptide (100 μl total volume), directly mixed by pipetting up and down five times and then immediately transferred to cells. All pipetting steps were done in a laminar flow cell culture hood and standardized to 15 min for each plate before transferring the plate of cells to a 37 °C CO_2_ incubator for 1.5 hr before addition of 500 μl DMEM supplemented with 10% FBS and another incubation for 3.5 hr in the 37 °C CO_2_ incubator (alternatively cells were incubated 2 h with peptides and another 4 hr with DMEM, 10% FBS (Fig. S1b–d). For the transduction time-course, cells were incubated with peptides in transduction buffer until indicated time-points. For inhibition of macropinocytosis, MDA-MB-231c#G3 were first pre-treated with 80 μM EIPA or Vehicle (DMSO), then transduced with 60 μM GFPβ11-TAT with 80 μM EIPA or Vehicle (DMSO) for 40 or 80 min before being analyzed. All cells were trypsinized and collected in 250 μl OptiMEM without phenol red and analyzed by FACS (GFP, FSC/SSC). 4,000 viable cells were analyzed per sample. Data is presented as fold change in fluorescence compared to non-treated cells. Video microscopy was performed using a Zeiss fluorescent microscope with imaging starting 15 min after addition of control GFPβ11 plus TAT peptides (*in trans*) (top panel) and GFPβ11-TAT peptide (bottom panel) to GFPβ1-10 H1299c#G3 cells and concluded at 140 min post-addition.

### Cell morphology and cell viability

Cell morphology was determined by FACS analysis of FSC and SSC. Gates were set manually for viable cells using untreated control cells as reference and the fraction of viable cells compared to non-viable cells was determined for each sample. Data are presented as the relative difference compared to non-treated control cells. Viable cells per sample were determined by measuring number of viable cells that were analyzed per second by FACS. Gates for viable cells were set manually using untreated control cells as reference. Data are presented as the relative difference compared to untreated control cells.

### Macropinocytosis assay

3,000 H1299 G1-10 cells were plated in a 48-well plate and treated with 10 μM TAT-P6-GG, TAT-P6-GWWG or TAT-P6-GFWFG peptides for 70 min in the presence of 70-kDa neutral dextran-Texas Red (100 μg/mL; Invitrogen) at 37° in triplicate. Cells were washed, trypsinized and analyzed by FACS (>2,000 cells per sample).

## Additional Information

**How to cite this article**: Lönn, P. *et al.* Enhancing Endosomal Escape for Intracellular Delivery of Macromolecular Biologic Therapeutics. *Sci. Rep.* **6**, 32301; doi: 10.1038/srep32301 (2016).

## Supplementary Material

Supplementary Information

Supplementary Figure S4
