# Supplementary material for: Enhancing Endosomal Escape for Intracellular Delivery of Macromolecular Biologic Therapeutics
Source: Sci Rep. 2016 Sep 8;6:32301. doi: 10.1038/srep32301 (PMC5015074; doi:10.1038/srep32301)
Supplement: Supplementary Information [file srep32301-s1.pdf]

Supplementary Materials

Enhancing Endosomal Escape for Intracellular Delivery of Macromolecular Biologic Therapeutics

Peter Lönn[1,2], Apollo D. Kacsinta[1], Xian-Shu Cui[1], Alexander S. Hamil[1], Manuel Kaulich[1,3], Khirud Gogoi[1,4] and Steven F. Dowdy[1]*

[1]Dept of Cellular and Molecular Medicine, UCSD School of Medicine, La Jolla, CA, USA, 92093

*Corresponding author (email: sdowdy@ucsd.edu)

Supplementary Figures

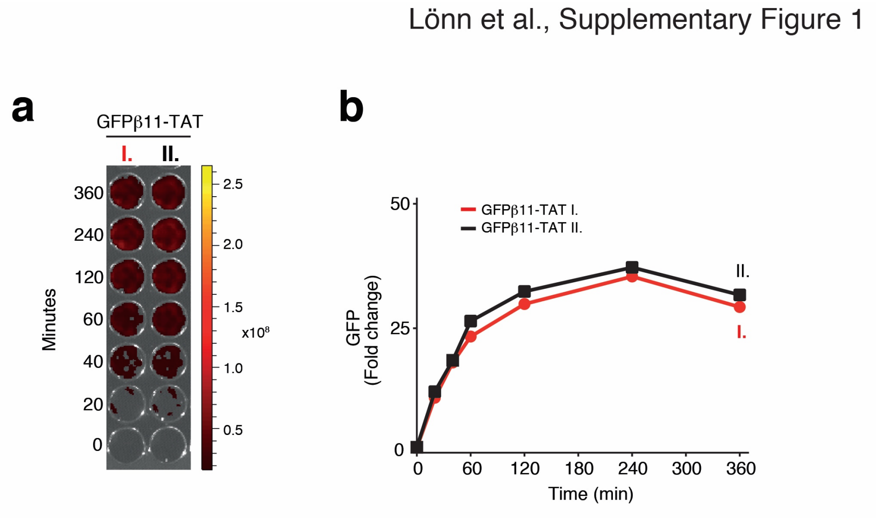

Supplementary Figure S1. Maturation of recombinant GFPβ11-TAT/GFP-β1-10 at 37 ^{O}C. (**a, b**) GFPβ11-TAT was incubated together with GFPβ1-10 in PBS for different time-points at 37 ^{O}C before being imaged on an IVIS Spectrum (**a**) and plotted as a graph of fold GFP fluorescence over background (**b**). The graph displays single sample measurements. Duplicate samples were compared (I, II).

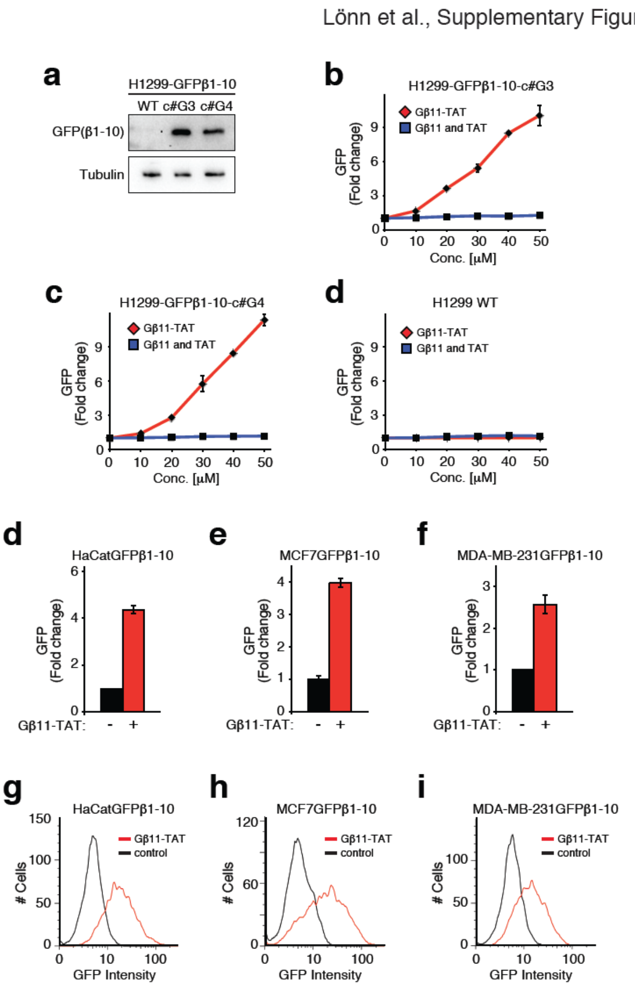

Supplementary Figure S2. Characterization of GFPβ1-10 expressing H1299c#G3/c#G4 human lung adenocarcinoma cells. (**a**) Western blot of expression levels of GFPβ1-10 in H1299, H1299c#G3, and H1299c#G4 cells. Blotting with anti-tubulin was used as loading control. (**b-d**) Dose-dependent comparison of H1299, H1299c#G3, and H1299c#G4 human lung adenocarcinoma cells treated with GFPβ11-TAT peptide or control GFPβ11 peptide plus control TAT peptide (*in trans*) analyzed by FACS. Graphs display mean values of triplicate samples with S.D. (**d-i**) FACS analysis of GFPβ1-10 expressing HaCaT (**d,g**), MCF7 (**e,h**), and MDA-MB-231 (**f,i**) cells treated with 60 μM GFPβ11-TAT peptide or untreated control. Bar graphs represent mean values of triplicate FACS samples with S.D.

Lönn et al., Supplementary Figure 3

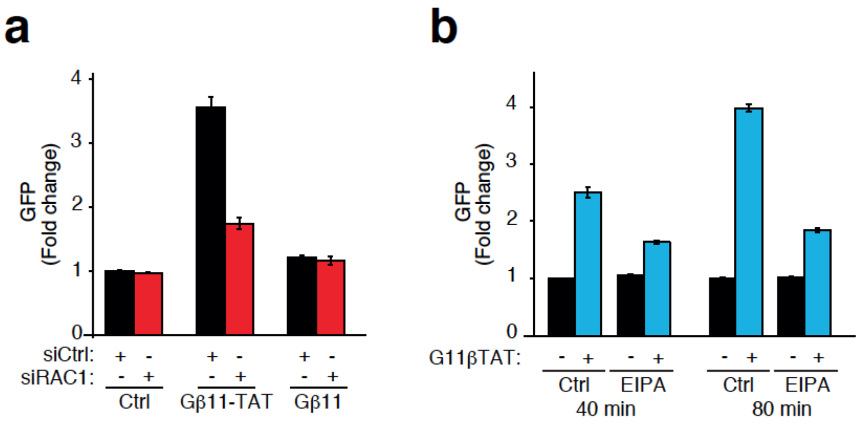

Supplementary Figure S3. Inhibiting macropinocytosis blocks GFPβ11-TAT cellular uptake. (**a**) GFPβ1-10 expressing H1299 cells were transfected with siCTRL or siRAC1 before being treated with 20 μM or 40 μM GFPβ11-TAT or 40 μM GFPβ11 peptide as control. Samples were measured for GFP fluorescence on FACS. The bar graphs show mean values of triplicate samples with S.D. (**b**) GFPβ1-10 expressing MDA-MB-231 were pretreated with or without 80 μM EIPA before being transduced with 60 μM GFPβ11-TAT with or without 80 μM EIPA for 40 min or 80 min and assayed for GFP fluorescence on FACS. DMSO was used as vehicle control. The bar graphs show mean values of triplicate samples with S.D.

Supplementary Figure S4. Fluorescence video microscopy of GFPβ11-TAT complementation of GFPβ1-10 cellular uptake kinetics. Control GFPβ11 plus TAT peptides (*in trans*) (top panel) and GFPβ11-TAT peptide (bottom panel) (80 μM) were added to GFPβ1-10 expressing H1299 cells and analyzed over time by fluorescence video microscopy from 15 min to 100 min post-addition.

Lönn et al., Supplementary Figure 5

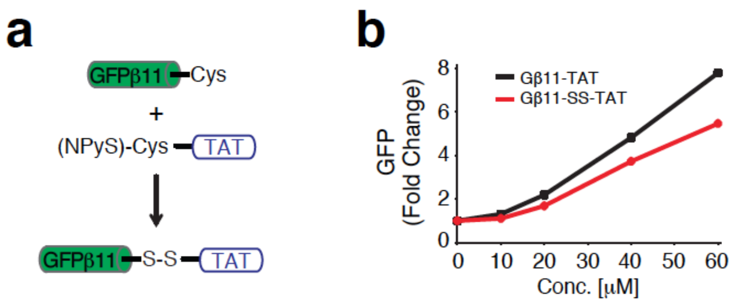

Supplementary Figure S5. (**a**) Structure and conjugation approach to generate GFPβ11-(S-S)-TAT peptide. (**b**) Dose-dependent comparison of GFPβ1-10 H1299 cells treated with parental GFPβ11-TAT peptide compared to GFPβ11-(S-S)-TAT peptide by FACS for GFP fluorescence.

Lönn et al., Supplementary Figure 6

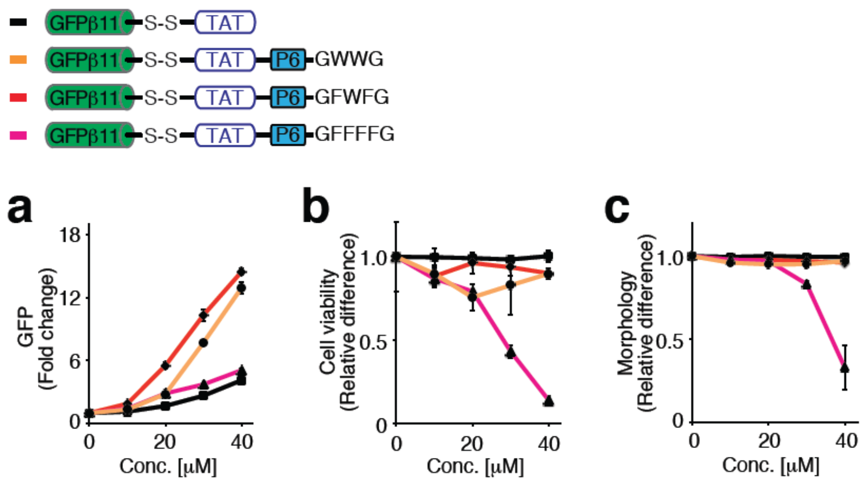

Supplementary Figure S6. Dose-dependent comparison of GFPβ1-10 expressing H1299 cells treated with GFPβ11-(S-S)-TAT-(EED) peptides containing four aromatic ring hydrophobic residues PEG6-GFWFG, PEG6-GWWG, or PEG6-GFFFFG, to parental GFPβ11-(S-S)-TAT peptide by FACS for GFP fluorescence (**a**), cellular morphology (**b**) and cell viability (**c**). The graphs display mean values of triplicate samples with S.D.

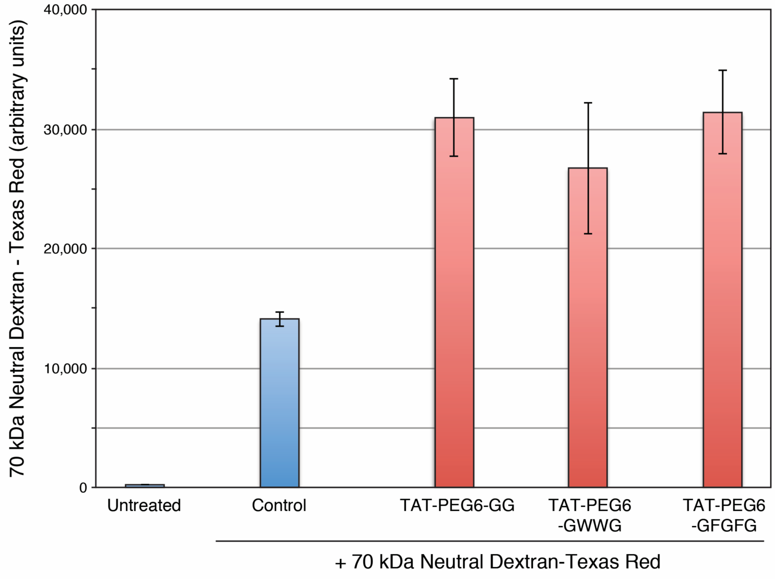

Supplementary Figure S7. Comparison of TAT-PEG6-GG, TAT-PEG6-GFWFG and TAT-PEG6-GWWG peptides (10 μM) to stimulate macropinocytosis vs. control. H1299 GFPβ1-10 cells were treated with each peptide for 70 min in the presence of the 70 kDa neutral dextran-texas red macropinocytosis marker and assayed by FACS. The graph displays mean values of triplicate samples with S.D.
